# Clinical characteristics and outcomes in women and men hospitalized for coronavirus disease 2019 in New Orleans

**DOI:** 10.1186/s13293-021-00359-2

**Published:** 2021-02-05

**Authors:** Yilin Yoshida, Scott A. Gillet, Margo I. Brown, Yuanhao Zu, Sarah M. Wilson, Sabreen J. Ahmed, Saritha Tirumalasetty, Dragana Lovre, Marie Krousel-Wood, Joshua L. Denson, Franck Mauvais-Jarvis

**Affiliations:** 1 Section of Endocrinology and Metabolism, Deming Department of Medicine, Tulane University School of Medicine, 1430 Tulane Ave. SL53, New Orleans, LA 70112 USA; 2 Southeast Louisiana Veterans Affairs Healthcare System, New Orleans, LA 70119 USA; 3 Deming Department of Medicine, Tulane University School of Medicine, 1430 Tulane Ave. SL53, New Orleans, LA 70112 USA; 4 Department of Biostatistics and Data Science, Tulane University School of Public Health and Tropical Medicine, 1430 Tulane Ave. SL53, New Orleans, LA 70112 USA; 5 Department of Epidemiology, Tulane University School of Public Health and Tropical Medicine, 1430 Tulane Ave. SL53, New Orleans, LA 70112 USA; 6 Section of Pulmonary Diseases, Critical Care, and Environmental Medicine, Deming Department of Medicine Tulane University School of Medicine, 1430 Tulane Ave. SL53, New Orleans, LA 70112 USA

## Abstract

**Objectives:**

Determine if sex differences exist in clinical characteristics and outcomes of adults hospitalized for coronavirus disease 2019 (COVID-19) in a US healthcare system.

**Design:**

Case series study.

**Setting and participants:**

Sequentially hospitalized adults admitted for COVID-19 at two tertiary care academic hospitals in New Orleans, LA, between 27 February and 15 July 2020.

**Measures and outcomes:**

Measures included demographics, comorbidities, presenting symptoms, and laboratory results. Outcomes included intensive care unit admission (ICU), invasive mechanical ventilation (IMV), and in-hospital death.

**Results:**

We included 776 patients (median age 60.5 years; 61.4% women, 75% non-Hispanic Black). Rates of ICU, IMV, and death were similar in both sexes. In women versus men, obesity (63.8 vs 41.6%, *P* < 0.0001), hypertension (77.6 vs 70.1%, *P* = 0.02), diabetes (38.2 vs 31.8%, *P* = 0.06), chronic obstructive pulmonary disease (COPD, 22.1 vs 15.1%, *P* = 0.015), and asthma (14.3 vs 6.9%, *P* = 0.001) were more prevalent. More women exhibited dyspnea (61.2 vs 53.7%, *P* = 0.04), fatigue (35.7 vs 28.5%, *P* = 0.03), and digestive symptoms (39.3 vs 32.8%, *P* = 0.06) than men. Obesity was associated with IMV at a lower BMI (> 35) in women, but the magnitude of the effect of morbid obesity (BMI ≥ 40) was similar in both sexes. COPD was associated with ICU (adjusted OR (aOR), 2.6; 95%CI, 1.5–4.3) and IMV (aOR, 1.8; 95%CI, 1.2–3.1) in women only. Diabetes (aOR, 2.6; 95%CI, 1.2–2.9), chronic kidney disease (aOR, 2.2; 95%CI, 1.3–5.2), elevated neutrophil-to-lymphocyte ratio (aOR, 2.5; 95%CI, 1.4–4.3), and elevated ferritin (aOR, 3.6; 95%CI, 1.7–7.3) were independent predictors of death in women only. In contrast, elevated D-dimer was an independent predictor of ICU (aOR, 7.3; 95%CI, 2.7–19.5), IMV (aOR, 6.5; 95%CI, 2.1–20.4), and death (aOR, 4.5; 95%CI, 1.2–16.4) in men only.

**Conclusions:**

This study highlights sex disparities in clinical determinants of severe outcomes in COVID-19 patients that may inform management and prevention strategies to ensure gender equity.

**Supplementary Information:**

The online version contains supplementary material available at 10.1186/s13293-021-00359-2.

## Introduction

Severe acute respiratory syndrome coronavirus 2 (SARS-CoV-2), which causes coronavirus disease 2019 (COVID-19), is disproportionately impacting older subjects with chronic medical comorbidities, and men, reportedly, exhibit a uniformly more severe outcomes than women [1, 2]. In case series from China, Europe, and the USA, COVID-19 hospitalizations, admission to intensive care unit (ICU), and in-hospital death have consistently been higher in men than in women [1–6]. The reasons for this sex disparity in COVID-19 outcomes are not entirely understood. Most diseases are characterized by sex differences in clinical presentation, evolution, and response to treatment [5]. Characterizing differences between men and women in COVID-19 presentation and outcomes is a central consideration in clinical research, as it may open therapeutic avenues to promote health equity in COVID-19 severity. To date, the sex-stratified analysis of clinical and biological characteristics of COVID-19 patients in relation to outcomes is still not available from most studies. New Orleans, LA, was an early epicenter, with the highest death rate per-capita in the USA noted during the April 2020 peak of the outbreak [7]. In this population, we reported that the high prevalence of metabolic disease including hypertension, obesity, and diabetes, dramatically increased the odds of mortality [7]. Further, in contrast to prior studies, our hospitalized population exhibited a predominance of women. These early observations led to the hypothesis that sex disparities may, in part, account for the greater severity of illness seen among this disadvantaged population suffering worse outcomes from COVID-19. This case series describes the association of sex with clinical characteristics and outcomes in 776 consecutive women and men hospitalized with COVID-19 in two tertiary care academic hospitals in urban New Orleans from 27 February to 15 July 2020.

## Methods

### Design and data source

This is a retrospective case series using data from 776 adult patients consecutively admitted for COVID-19 at two tertiary care academic hospitals, Tulane Medical Center and University of Medical Center in New Orleans, LA, from 27 February to 15 July 2020. These hospitals serve racially diverse, low-income populations across metropolitan New Orleans. All adults (> 18 years) hospitalized with confirmed SARS-CoV-2 (COVID-19) infection on admission were included [COVID-19 infection assessed by polymerase chain reaction of a nasopharyngeal sample] and were subsequently hospitalized. In-hospital death or discharge status was assessed through 23 July 2020. This study was reviewed and approved with waiver of consent by the Tulane University Biomedical Institutional Review Board (IRB) and the University Medical Center New Orleans Research Review Council.

### Data collection and measurement

Demographic and clinical data were extracted from two hospitals’ medical records. Six clinicians then performed manual medical record review to organize and verify the data, with discrepancies resolved following discussion with two senior investigators and attending physicians. This dataset included the following domains: demographic characteristics (age at admission, sex, patient-reported race, and hospital site), clinical symptoms at admission, comorbidities, laboratory values at or right after admission, and COVID-19 outcomes including ICU admission, invasive mechanical ventilation (IMV), and in-hospital mortality. Comorbid conditions, including chronic obstructive pulmonary disease (COPD), asthma, cardiovascular disease (CVD), cerebrovascular disease, chronic kidney disease (CKD), chronic liver disease (CLD), and dementia were ascertained by codes in the International Classification of Diseases, 10th Revision [ICD-10] and physician notes 6 months prior to the admission. Diabetes and pre-diabetes were defined by documented diagnosis, elevated hemoglobin A1c value, or the use of anti-diabetic medications. Hypertension and hyperlipidemia were defined by documented diagnoses or use of antihypertensive or lipid-lowering medications.

### Statistical analysis

To compare patients’ characteristics at admission by sex, we used chi-square test (Fisher’s exact test when appropriate) for categorical variables and two tailed *t* test for continuous variables. To describe the relationship between comorbidities, biomarkers, and outcomes in the overall sample, we performed univariate and then multivariable logistic regressions adjusting for age, sex, hospital site, and the Charlson Comorbidity Index [8]. Sex (women vs men) and race (non-Hispanic Black vs non-Black) stratified analyses were also performed separately. All model-based results are presented with 95% confidence intervals. All analyses were conducted with the use of the SAS System for Windows, version 9.4 (SAS Institute).

## Results

### Sex differences in baseline characteristics of the study population

The overall baseline characteristics (overall and by sex) of our cohort are presented in Table 1 and race-specific baseline characteristics in eTable 1. Mean age of the cohort was 60.5 years, 61.4% were women, and the majority of participants (583; 75%) self-identified as Black. Black patients were slightly younger than non-Black patients. While Black women and men exhibited no age difference, non-Black women were older than non-Black men (eTable 1). White women were significantly older than White men (71.7 vs 63.7-year-old, *P* = 0.03) and Black women (71.7 vs 60.2-year-old, *P* < 0.0001, results not shown in tables). The most common comorbid conditions were hypertension (74%), obesity (53%), and diabetes (35%), followed by CVD (21%), COPD (19%), CKD (17%), and asthma (11%). A non-obese (< 30 kg/m^2^) or normal BMI (< 24.9 kg/m^2^) was more common in men than women, whereas obesity (BMI ≥ 30 kg/m^2^) was more common in women than in men, with morbid obesity (BMI > 40 kg/m^2^) being over twice as prevalent among women as men (Table 1). Similarly, hypertension, COPD, asthma, and to a lesser extent, diabetes were more common at baseline among women than men, while CLD was more common among men (Table 1). When data were analyzed by race, the prevalence of all comorbidities was higher in Black compared to non-Black patients (eTable 1). A similar sex difference was observed for obesity, asthma and CLD in the Black, as in the overall sample (eTable 1). In the Black sample, women were more likely to have obesity and asthma at baseline, while men were more likely to have CKD and CLD. In non-Black patients, women had higher prevalence of hypertension, obesity, and dementia compared to men (eTable 1). No sex difference was observed in the prevalence of other comorbidities in either Black or non-Black samples. Non-Black, and especially White women, had significantly higher Charlson Comorbidity Index score than Black women (mean index, 4.8 vs 3.7, *P* = 0.01, results not shown in tables).

**Table 1:** Demographic characteristics and comorbidities prior to admission

	All	Women	Men	***P*** value
**Age, mean (SD), years**	60.5 (16.1)	61.4 (16.7)	59.8 (15.2)	0.16
	***N*, (%)**	***N*, (%)**	***N*, (%)**	
**Sex**	**---**	406 (52.7)	365 (47.3)	0.14
**Race**				0.16
White	99 (12.7)	45 (11.1)	54 (14.8)	
Black	583 (75)	315 (77.6)	261 (71.7)	
Other	95 (12.3)	46 (11.3)	49 (13.5)	
**Hospital site**				0.87
Tulane	298 (38.4)	158 (38.9)	140 (38.4)	
UMC ^a^	478 (61.6)	248 (61.1)	225 (61.6)	
**Comorbidity**				
Obesity	409 (53.1)	257 (63.8)	151 (41.6)	**< 0.0001**
BMI (kg/m^2^)^b^ category				**< 0.0001**
BMI < 25	164 (21.3)	69 (17.1)	93 (25.6)	
25 ≤ BMI < 30	198 (25.7)	77 (19.1)	119 (32.8)	
30 ≤ BMI < 35	157 (20.4)	89 (22.1)	68 (18.7)	
35 ≤ BMI < 40	118 (15.3)	76 (18.9)	42 (11.6)	
BMI ≥ 40	134 (17.4)	92 (22.8)	41 (11.3)	
Diabetes	273 (35.2)	155 (38.2)	116 (31.8)	0.06
Hypertension	573 (73.8)	315 (77.6)	256 (70.1)	**0.02**
Dyslipidemia	291 (37.5)	158 (38.9)	132 (36.2)	0.4
Chronic obstructive pulmonary disease	140 (18.8)	87 (22.1)	52 (15.1)	**0.015**
Asthma	83 (10.7)	58 (14.3)	25 (6.9)	**0.001**
Cerebrovascular disease	94 (12.6)	51 (12.9)	43 (12.5)	0.84
Cardiovascular disease	154 (20.7)	84 (21.3)	70 (20.3)	0.73
Heart failure	109 (14.7)	62 (15.7)	47 (13.6)	0.42
Myocardial infarction	46 (6.2)	20 (5.1)	26 (7.5)	0.17
Peripheral vascular disease	25 (3.4)	16 (4.1)	9 (2.6)	0.27
Chronic kidney disease	126 (16.9)	62 (15.7)	64 (18.6)	0.31
Chronic liver disease	36 (4.6)	11 (2.7)	25 (6.9)	**0.007**
Dementia	55 (7.4)	34 (8.6)	20 (5.8)	0.14
**Charlson Index, mean (SD)**	3.7 (2.8)	3.8 (2.7)	3.7 (2.7)	0.68

^a^*UMC* University Medical Center New Orleans

^b^*BMI* body mass index

### Sex differences in clinical symptoms and biomarkers at admission

At admission, women were more likely than men to present with digestive symptoms, dyspnea, and fatigue (Table 2), and this phenotype was driven by Black women (eTable 1). In Black patients, more women reported non-productive cough than men. In non-Black patients, more men had fever at admission than women (eTable 1).

**Table 2:** Clinical symptoms and biomarkers at admission

	All (***N*** [%])	Women (***N*** [%])	Men (***N*** [%])	***P*** value
**Clinical symptoms**				
Fever	436 (59.3)	223 (57.3)	210 (61.6)	0.24
Headache	39 (5.3)	25 (6.4)	13 (3.8)	0.11
Nausea/vomiting	154 (21)	99 (25.5)	54 (15.8)	**0.001**
Diarrhea	187 (25.4)	104 (26.7)	80 (24.5)	0.31
Digestive symptoms (nausea, vomiting, or diarrhea)	269 (36.6)	153 (39.3)	112 (32.8)	**0.06**
Abdominal pain	65 (8.8)	37 (9.5)	28 (8.2)	0.54
Rhinorrhea	28 (3.8)	18 (4.6)	10 (2.9)	0.23
Sore throat	47 (6.4)	25 (6.4)	21 (6.2)	0.88
Cough (productive)	125 (17)	62 (16)	63 (18.5)	0.36
Cough (no-productive)	329 (44.8)	184 (47.3)	143 (41.9)	0.15
Chest tightness or pain	99 (13.5)	53 (13.6)	46 (13.5)	0.95
Dyspnea	422 (57.4)	238 (61.2)	183 (53.7)	**0.04**
Anosmia	18 (2.5)	10 (2.6)	8 (2.4)	0.84
Ageusia	25 (3.4)	13 (3.3)	12 (3.5)	0.9
Myalgia	203 (27.7)	115 (29.6)	87 (25.5)	0.21
Confusion	127 (17.3)	65 (16.7)	62 (18.2)	0.6
Fatigue	237 (32.3)	139 (35.7)	97 (28.5)	**0.03**
**Biomarkers**				
ALT > 56 U/L ^a^ (*n* = 729)	204 (278)	54 (21.4)	88 (36.8)	**0.0002**
AST >40 U/L ^b^ (*n* = 727)	192 (26.4)	47 (18.7)	88 (36.9)	**< 0.0001**
CRP > 3 mg/L ^c^ (*n* = 641)	632 (98.6)	270 (98.2)	264 (97.8)	0.73
D-dimer > 0.5 mg/L (*n* = 541)	486 (89.8)	147 (63.1)	131 (57.2)	0.2
Ferritin > 300 ng/mL (*n* = 640)	437 (68.3)	163 (58.8)	199 (75.4)	**< 0.0001**
Glucose ≥ 140 mg/dL (*n* = 598)	286 (48.6)	153 (47.7)	130 (49.1)	0.74
A1c > 5.7% (*n* = 262)	262 (83.7)	163 (80.7)	143 (78.1)	0.53
LDH ≥ 220 U/L^d^ (*n* = 629)	522 (83)	226 (81.3)	213 (82.9)	0.63
Procalcitonin ≥ 0.1 ng/mL (*n* = 494)	423 (85.6)	98 (56.7)	112 (69.6)	**0.01**
Troponin ≥ 0.04 ng/ml (*n* = 549)	121 (22)	62 (29.7)	53 (28.3)	0.77
NT-Pro BNP > 400 pg/ml ^e^ (*n* = 603)	178 (29.5)	39 (41.5)	27 (38)	0.65
WBC count < 4.0 × 10^3^/uL ^f^ (*n* = 732)	71 (9.7)	20 (9.4)	22 (11.2)	0.55
Lymphocytes < 1.0 × 10^3^/uL (*n* = 732)	304 (41.5)	74 (36.8)	86 (46.5)	0.06
Platelets < 150 × 10^3^/uL (*n* = 733)	121 (16.5)	36 (17.9)	35 (18.9)	0.8
Neutrophils > 1.8 × 10^3^/uL (*n* = 732)	709 (96.9)	199 (99.5)	182 (100)	0.34
NLR>6 ^g^ (*n* = 732)	275 (37.6)	125 (32.1)	147 (43.4)	**0.001**
Monocytes > 1.0 × 10^3^/uL (*n* = 732)	104 (14.2)	27 (12.8)	37 (18.9)	0.09

^a^*ALT* alanine aminotransferase

^b^*AST* aspartate aminotransferase

^c^ *CRP* C-reactive protein

^d^*LDH* lactate dehydrogenase

^e^*NT-Pro BNP* N-terminal pro b-type natriuretic peptide

^f^*WBC* white blood cell counts

^g^*NLR* neutrophil-to-lymphocyte ratio

A greater proportion of men than women had ALT, AST, procalcitonin, ferritin, and neutrophil-to-lymphocyte ratio (NLR) above the normal range (Table 2); results were qualitatively similar in the Black sample who additionally showed a greater percentage of monocytes (eTable 2). In non-Black patients, more men than women had elevated AST and ferritin (eTable 2).

During hospitalization, 271 patients (34.9%) were admitted to ICU, among which 144 were women and 125 men were (35.5 vs 34.3%, *P* = 0.7), 187 patients (24.1%) received IMV, among which 100 were women and 85 were men (24.6 vs 23.3%, *P* = 0.7). In-hospital death occurred in 140 patients (18.1%), among which 71 were women and 66 were men (17.5 vs 18.1%, *P* = 0.8). Black versus non-Black patients had higher incidence of ICU admission (37.7 vs. 26.3%, *P* = 0.004) and IMV (26.5 vs. 17%, *P* = 0.007); in-hospital death rate for Black (106 patients, 18.3%) vs White patients (17 patients, 17.1%) was not statistically significant (*P* = 0.9). Results were similar for each comparison in women vs men within each racial group (*P* > 0.05—data not shown in tables).

### Multivariable analyses of comorbidities with outcomes

**Fig. 1:**
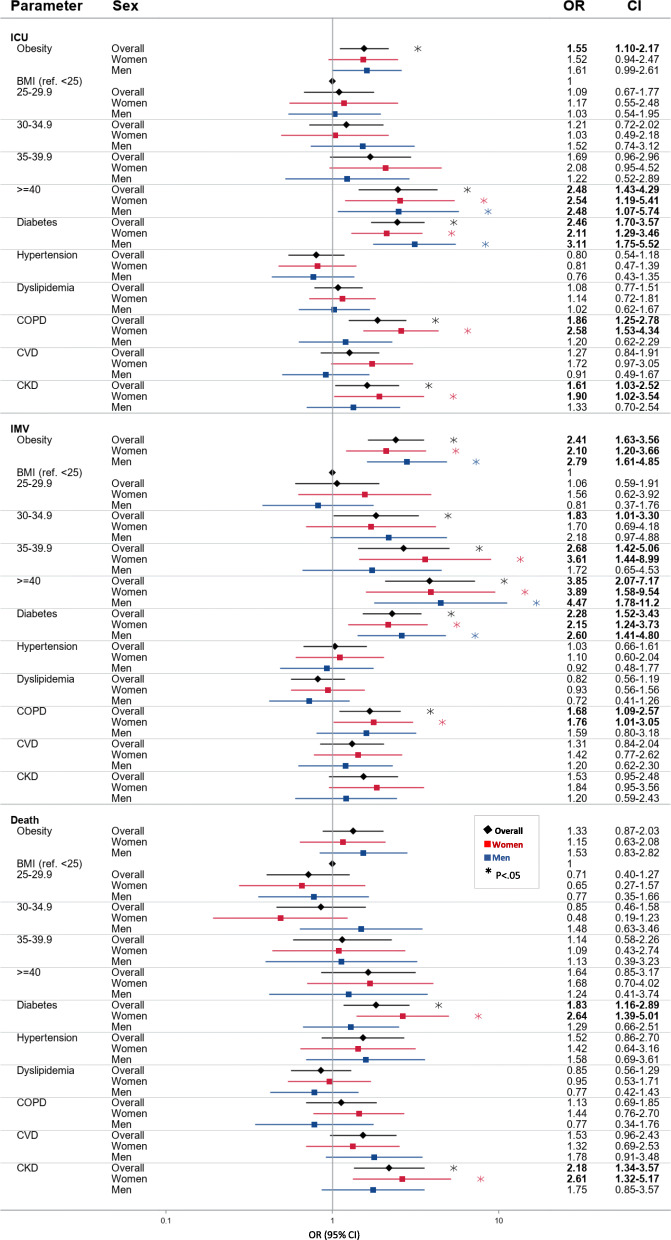
Multivariable analysis of Comorbidities and Outcomes

In adjusted multivariable analyses, obesity was independently associated with increased odds of IMV and ICU admission in the overall sample (Fig. 1). When data were stratified by sex, only morbid obesity (BMI ≥ 40 kg/m^2^) was independently associated with increased odds of ICU admission in both women and men (Fig. 1). Obesity was independently associated with increased odds of IMV at a lower BMI (> 35 kg/m^2^) in women than men, but the magnitude of the effect of morbid obesity (BMI ≥ 40 kg/m^2^) was similar in both sexes (Fig. 1). When multivariable analyses were performed by race, obesity was associated with increased odds of ICU, IMV, and death in the overall Black patients, which remained significant for IMV in Black women and men (eFigure 1). However, obesity was not associated with death in the non-Black women and men (eFigure 1). In adjusted analyses, diabetes was independently associated with increased odds of ICU admission and IMV in the overall sample, and in women and men (Fig. 1). Similar results were observed for ICU and IMV in race-specific analyses (eFigure 1). Notably, diabetes was independently associated with increased odds of in-hospital death in the overall sample. When data were stratified by sex, diabetes was associated with increased odds of death in women, but not in men (Fig. 1). When stratifying by race, diabetes remained positively associated with increased odds of death in Black and non-Black women, not in Black and non-Black men (eFigure 1). The existence of COPD was independently associated with increased odds of ICU admission and IMV in women but not in men (Fig. 1), which remained significant for ICU in the Black sample, especially for Black women (eFigure 1). Notably, CKD was positively associated with the odds of ICU admission and death in the overall and women only sample. When data was disaggregated by race, CKD was associated with increased odds of ICU, IMV, and death in the overall Black patients (eFigure 1). However, CKD was associated with increased odds of in-hospital death in Black women only (eFigure 1).

### Multivariable analyses of biomarkers with outcomes

Several biomarkers of inflammation and hypercoagulability, including C-reactive protein (CRP) [9], N-terminal pro-type natriuretic peptide (NT-proBNP) [10], Lactate dehydrogenase (LDH) [11, 12], procalcitonin [13, 14], NLR [12, 15], ferritin [11], troponin [16, 17], and D-dimer [18] have been associated with COVID-19 severity.

**Fig. 2:**
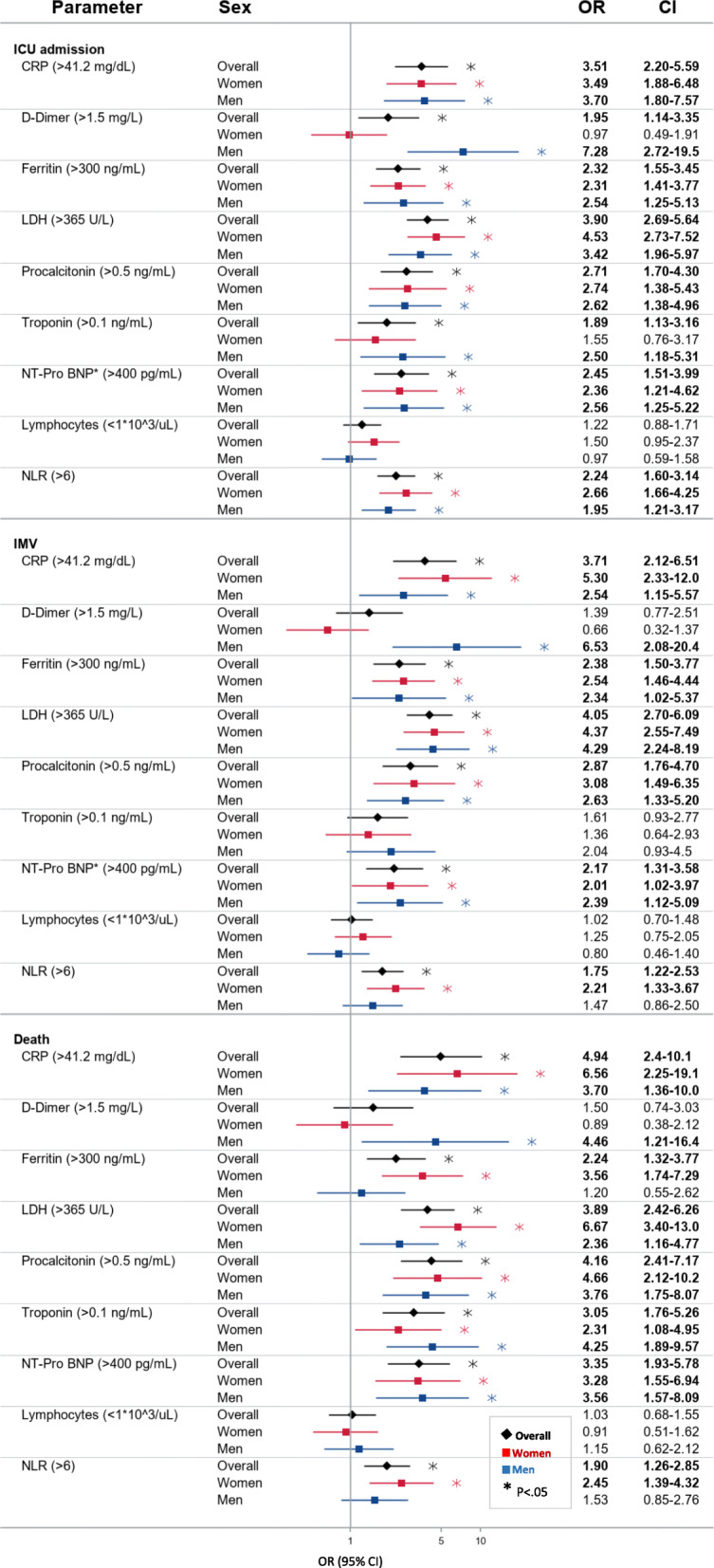
Multivariable analysis of Biomarkers and Outcomes

*CRP* is an acute-phase protein produced by the liver during inflammation following interleukin-6 secretion by macrophages, T cells, and adipocytes. CRP was similarly elevated in men and women in the overall and race-specific samples (Table 2 and eTable 1). In multivariable analyses, CRP was associated with increased odds of IMV, ICU, and death in the overall sample as well as in sex-specific samples (Fig. 2 and eFigure 2). When analyzed by race, similar results were observed in Black patients overall and by sex. The association between CRP, IMV, and ICU were found in the overall non-Black patients and non-Black women; whereas the association between CRP and death was found in the overall non-Black patients and non-Black men (eFigure 2).

*NT-proBNP* is a BNP prohormone released from the heart during heart failure. NT-proBNP was shown to be an independent risk factor for in-hospital death in patients with severe COVID-19 [19]. NT-proBNP was similarly elevated in men and women in the overall sample and in race-specific samples (Table 2 and eTable 1). NT-proBNP was independently associated with increased odds of ICU, IMV, and death in both sexes (Fig. 2). When stratifying race, the association of NT-proBNP with the ICU and IMV remained significant in Black patients overall. NT-proBNP was positively associated with the odds in-hospital death in Black men and women, and non-Black women (eFigure 2).

*LDH* rises following multiple organ injury and failure with decreased oxygenation. The elevation of LDH was similar in women and men in the overall sample, and in Black and non-Black sample, separately (Table 2 and eTable 1). Elevated LDH was independently associated with increased odds of IMV, ICU, and death in the overall sample (Fig. 2). When analyzed by sex, the magnitude of the effect of LDH on ICU and death was greater in women than in men (Fig. 2). Similar results were observed in Black and non-Black patients, separately (eFigure 2). *Procalcitonin* increases during infection and inflammation and is a marker of COVID-19 severity [13]. An elevated procalcitonin was more prevalent in men than in women in the overall and Black samples (Table 2 and eTable 1). Procalcitonin was independently associated with the increased odds of ICU admission, IMV, and death in patients overall (Fig. 2). When analyzed by sex, the magnitude of the association between procalcitonin and IMV and death was greater in women than in men (Fig. 2). This sex difference was also observed in Black patients (eFigure 2). Procalcitonin was also independently associated with increased odds of ICU admission, IMV, and death in the overall and sex-specific samples. The same association was also found in the overall non-Black sample (eFigure 2).

*Lymphopenia with increased neutrophils* is a criteria for severe COVID-19 and predicts the severity clinical outcomes [20]. A greater proportion of men exhibited an increased NLR > 6 than women with a trend toward increased prevalence of lymphocytopenia and increased monocytes compared to women (Table 2). When data was stratified by race, the male bias in lymphopenia and increased monocytes became significant in Black patients (eTable 1). The increased NLR was independently associated with increased odds of ICU, IMV, and death in patients overall (Fig. 2). When data were stratified by sex, the magnitude of the association between NLR and ICU admission and IMV was greater in women than in men (Fig. 2). Notably, the increased NLR was independently associated with death in women only, but not in men. When analyzed by race, of the association between NLR and ICU admission, IMV, and death remained significant in both Black and non-Black patients, and mainly driven by women (eFigure 2).

*Ferritin* is a key mediator of immune dysregulation via direct immune-suppressive and pro-inflammatory effects, contributing to the cytokine storm [21]. An elevated ferritin was more prevalent in men than in women in the overall and Black samples (Table 2 and eTable 1). Ferritin was independently associated with increased odds of ICU admission, IMV, and death in patients overall and by sex (Fig. 2). Similar results were observed in the Black patients (eFigure 2). However, ferritin was independently associated with death in women only in the overall and Black sample (Fig. 2 and eFigure 2).

Elevated cardiac *troponin* levels, a marker of myocardial injury, are associated with increased mortality in patients with COVID-19 [19, 22]. Troponin was similarly elevated in men and women in the overall sample (Table 2) but was more elevated in Black men than Black women (eTable 1). Troponin was independently associated with ICU admission and death in the overall sample (Fig. 2). When analyzed by sex, troponin was associated with increased odds of ICU admission only in men, and the magnitude of the effect of troponin on the odds of death was greater in men than in women (Fig. 2). When analyzed by race, troponin was independently associated with death in Black and non-Black men, but not in women (eFigure 2).

*D-dimer* is a marker of activated coagulation commonly elevated in patients with COVID-19, which correlates with disease severity [18]. D-dimer was similarly elevated in men and women in the overall and Black samples (Table 2 and eTable 1). Notably, D-dimer elevation was independently associated with increased odds of ICU admission, IMV, and death in men only, but not in women (Fig. 2). When disaggregating race, D-dimer elevation was positively associated with ICU admission and IMV in Black men, and ICU admission and death in non-Black men (eFigure 2).

## Discussion

This study highlights sex disparities in demographic characteristics, as well as clinical and biological presentation of the initial 776 sequential hospitalized adult patients with confirmed COVID-19 at two large urban medical centers in New Orleans.

The first finding of this study is that COVID-19 outcomes (hospitalization, admission to ICU, IMV, and in-hospital death) were similar in women and men. This contracts with most published case series from Asia, Europe, and the USA where men with COVID-19 had higher proportion of hospitalization and more severe outcomes compared to women [1–7, 23]. These findings may be unique to the predominantly Black patient population cared for at these urban centers, as other studies have found similar results in predominantly Black patients of New Orleans and Detroit [7, 23]. Supporting this hypothesis, our Black cohort contained a higher percentage of women than men. However, consistent with other case series, our non-Black cohort contained a greater proportion of men than women. Additionally, Black women exhibited a similar age and Charlson Comorbidity Index as Black men. In contrast, White women were older and exhibited a higher Charlson Comorbidity Index than White men and Black women. Women, and especially Black women, also exhibited greater proportion of comorbidities including obesity, hypertension, diabetes, COPD, and asthma than men. Therefore, and consistent with other studies, non-Black women seem to exhibit a biological or socio-demographic advantage compared to non-Black men regarding COVID-19 hospitalization in New Orleans, as they need to be older and accumulate a higher index of comorbidities to be hospitalized. In contrast, in this cohort, Black women seem to have lost their female biological advantage regarding COVID-19 severity compared to Black men. They are hospitalized at a similar age and index of comorbidities than Black men, and at a younger age with a higher index of comorbidities than non-Black women. These finding highlights the health disparity that seem to affect Black women in New Orleans. Note that our hospitals serve a population with low socio-economic background, with high rate of Medicaid and uninsured patients, and an average Charlson Comorbidity Index of 3.9. We also observed that more women presented with digestive symptoms and this was significant in Black women only.

The second finding is that some comorbidities at baseline are determinants of more severe outcomes in women than men. Obesity was a predictor of respiratory failure requiring IMV at a lower level of obesity (BMI 35–40 kg/m^2^) in women than men (BMI > 40 kg/m^2^). The presence of COPD was a determinant of ICU admission and respiratory failure requiring IMV in women, but not in men. Notably, diabetes was a major independent determinant of death in Black and non-Black women, but not in men. Accumulating evidence suggest that women who develop type 2 diabetes experience an earlier, greater, and more prolonged deterioration in metabolic homeostasis than men, including central obesity, insulin resistance, inflammation, hypercoagulability, dyslipidemia, and hypertension [24–28]. All these factors increase the risk of COVID-19 mortality. Similarly reported in other COVID-19 case series [29–31], in our overall cohort, CKD was an independent determinant of death. Notably, after stratifying by sex, CKD was an independent determinant of death in all and in Black women, but not in men. This contrasts with other investigations of sex differences in CKD mortality. For example, in the Chronic Renal Insufficiency Cohort Study, a large CKD cohort of around 4000 racially diverse men and women, after adjusting for demographic and clinical factors, women had lower risk of death than men [32]. Therefore, the biological and social factors explaining why CKD is an independent predictor of death from COVID-19 in women, and especially Black women, deserve investigation.

As observed in Wuhan [33], men were more likely to exhibit systemic inflammation compared to women, with increased procalcitonin, ferritin, a NLR > 6, and a greater percentage of monocytes. However, the increased NLR and ferritin were independent predictors of death in women, independent of race, but not in men. The increased NLR in severe COVID-19 reflects a depletion in lymphocytes, particularly cytotoxic T lymphocytes, coupled with an increase in neutrophils that produce proinflammatory cytokines [34]. Consistent with our findings, a study of patients hospitalized for COVID-19 in New Heaven, reported that higher levels of innate immune proinflammatory cytokines, like those produced by neutrophil, were associated with worse disease progression in women, but not in men [35]. Ferritin is also a marker of innate immune (macrophage) activation [36]. Together, these data suggest that the exaggerated innate immune response could be a greater predictor of COVID-19 severity in women than in men.

In contrast, D-dimer, a marker of hypercoagulability was an independent predictor of severe outcomes or death in men, independent of race, but not in women. Men are at higher risk of venous thromboembolism (VTE) than women [37, 38], including during COVID-19 [39]. Coagulopathies resulting in VTE and disseminated intravascular coagulation have been reported to be the primary cause of death in critical COVID-19 patients [10]. Therefore, D-dimers could be a greater predictor of lethal COVID-19 coagulopathy in men than in women.

The biological factors underlying these sex disparities in immune, inflammatory, and hypercoagulability markers deserve further investigation, as they may have implications for sex-based treatment and vaccination.

This study has several limitations. First, the study population only included patients within the New Orleans area, and the findings may not be generalizable to other populations. Second, the limited number of patients from some ethnic groups, such as Asian and Native Americans precluded a finer racial/ethnic stratification in the analysis. We split patients into two general racial groups—Black and non-Black. However, our sample reflects the racial distribution of COVID-19 in New Orleans, where Blacks have been disproportionately affected by the pandemic. Lastly, this is a case series study of hospitalized patients with confirmed COVID-19. It does not include a comparison of outcomes in patients not exposed to COVID-19. The observational nature of the study does not permit us to draw conclusions on causal relationships between comorbidities, biomarkers, and the COVID-19 outcomes.

## Perspectives and significance

This study highlights sex disparities in clinical and biological determinants of severe outcomes in patients hospitalized for COVID-19 in New Orleans. These determinants may be of clinical utility to healthcare providers for management tailored to women and men hospitalized with COVID-19.

## Supplementary Information

**Additional file 1: eFigure 1.A.** Multivariable Analysis of Comorbidities and ICU by Race. **eFigure 1.B.** Multivariable Analysis of Comorbidities and IMV by Race. **eFigure 1.C.** Multivariable Analysis of Comorbidities and Death by Race. **eFigure 2.A.** Multivariable Analysis of Biomarkers and ICU by Race. **eFigure 2.B.** Multivariable Analysis of Biomarkers and IMV by Race. **eFigure 2.C.** Multivariable Analysis of Biomarkers and Death by Race. **eTable 1.** Demographic Characteristics and comorbidities prior to admission– Blacks vs. non-Blacks. **eTable 2.** Clinical symptoms and biomarkers at admission – Blacks vs. non-Blacks.

## Data Availability

The datasets generated and/or analyzed during the current study are not publicly available due to confidential personal health information, but are available from the corresponding author on reasonable request.
