# Supplementary material for: Clinical characteristics and outcomes in women and men hospitalized for coronavirus disease 2019 in New Orleans
Source: Biol Sex Differ. 2021 Feb 5;12:20. doi: 10.1186/s13293-021-00359-2 (PMC7863061; doi:10.1186/s13293-021-00359-2)
Supplement: Supplementary file 1 — Additional file 1: eFigure 1.A. Multivariable Analysis of Comorbidities and ICU by Race. eFigure 1.B. Multivariable Analysis of Comorbidities and IMV by Race. eFigure 1.C. Multivariable Analysis of Comorbidities and Death by Race. eFigure 2.A. Multivariable Analysis of Biomarkers and ICU by Race. eFigure 2.B. Multivariable Analysis of Biomarkers and IMV by Race. eFigure 2.C. Multivariable Analysis of Biomarkers and Death by Race. eTable 1. Demographic Characteristics and comorbidities prior to admission– Blacks vs. non-Blacks. eTable 2. Clinical symptoms and biomarkers at admission – Blacks vs. non-Blacks. [file 13293_2021_359_MOESM1_ESM.pdf]

eFig 1.A Multivariable Analysis of Comorbidities and ICU by Race

Parameter	Race	Sex	OR	CI
**ICU**				
Obesity	Black	Overall	**1.58**	**1.07-2.32**
		Women	1.70	0.98-2.95
		Men	1.50	0.87-2.58
	Non-Black	Overall	1.38	0.66-2.89
		Women	1.00	0.35-2.85
		Men	1.96	0.69-5.53
Diabetes	Black	Overall	**2.23**	**1.47-3.39**
		Women	**2.06**	**1.17-3.62**
		Men	**2.45**	**1.31-4.57**
	Non-Black	Overall	**3.42**	**1.53-7.66**
		Women	2.06	0.72-5.88
		Men	**8.51**	**2.16-33.5**
Hypertension	Black	Overall	0.74	0.47-1.17
		Women	0.70	0.37-1.29
		Men	0.82	0.42-1.61
	Non-Black	Overall	1.00	0.44-2.26
		Women	1.44	0.44-4.64
		Men	0.75	0.22-2.51
Dyslipidemia	Black	Overall	1.24	0.85-1.79
		Women	1.31	0.79-2.19
		Men	1.13	0.65-1.97
	Non-Black	Overall	0.55	0.24-1.29
		Women	0.54	0.16-1.78
		Men	0.58	0.17-1.99
COPD	Black	Overall	**1.94**	**1.26-2.99**
		Women	**2.59**	**1.48-4.53**
		Men	1.24	0.61-2.52
	Non-Black	Overall	1.59	0.53-4.77
		Women	2.69	0.57-12.7
		Men	1.03	0.18-5.84
CVD	Black	Overall	1.10	0.69-1.74
		Women	1.18	0.63-2.23
		Men	1.01	0.52-1.98
	Non-Black	Overall	2.32	0.93-5.81
		Women	**8.54**	**2.04-35.8**
		Men	0.49	0.09-2.54
CKD	Black	Overall	**1.64**	**1.00-2.68**
		Women	1.65	0.82-3.32
		Men	1.66	0.82-3.32
	Non-Black	Overall	1.38	0.46-4.12
		Women	3.02	0.77-11.8
		Men	---	

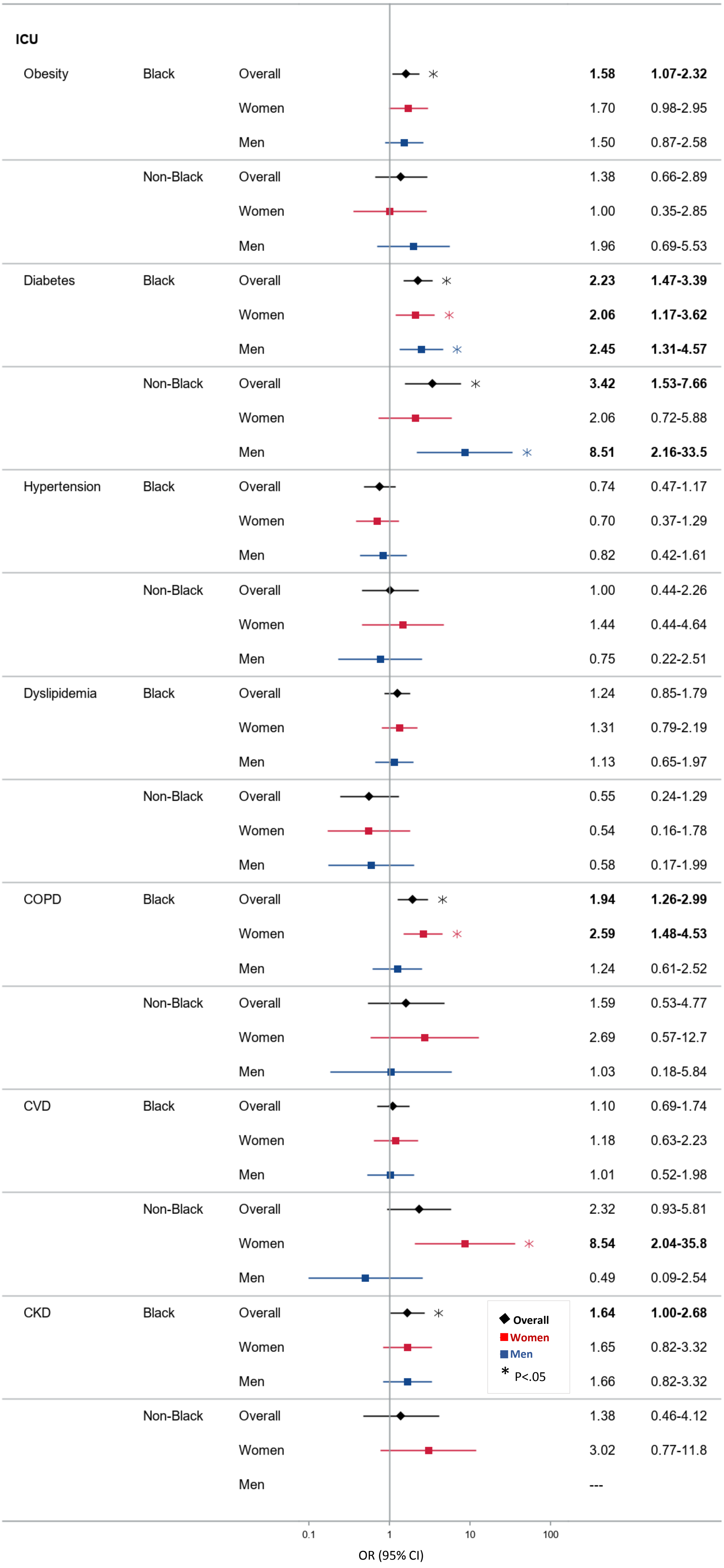

eFig 1.B Multivariable Analysis of Comorbidities and IMV by Race

Parameter	Race	Sex	OR	CI
**IMV**				
Obesity	Black	Overall	**2.73**	**1.76-4.24**
		Women	**2.38**	**1.26-4.48**
		Men	**3.12**	**1.69-5.76**
	Non-Black	Overall	1.51	0.62-3.66
		Women	1.26	0.36-4.36
		Men	1.69	0.45-6.36
Diabetes	Black	Overall	**1.91**	**1.21-3.00**
		Women	1.84	0.98-3.45
		Men	**2.06**	**1.07-3.99**
	Non-Black	Overall	**5.02**	**1.98-12.6**
		Women	**4.46**	**1.31-15.1**
		Men	**7.81**	**1.56-39.1**
Hypertension	Black	Overall	1.00	0.60-1.65
		Women	0.89	0.45-1.75
		Men	1.14	0.53-2.41
	Non-Black	Overall	1.11	0.41-2.99
		Women	2.57	0.58-11.3
		Men	0.32	0.06-1.58
Dyslipidemia	Black	Overall	0.89	0.59-1.34
		Women	1.00	0.57-1.74
		Men	0.80	0.43-1.46
	Non-Black	Overall	0.48	0.16-1.40
		Women	0.57	0.13-2.39
		Men	0.42	0.08-2.17
COPD	Black	Overall	**1.67**	**1.06-2.64**
		Women	1.73	0.97-3.10
		Men	1.55	0.74-3.25
	Non-Black	Overall	1.97	0.55-7.03
		Women	1.76	0.29-10.6
		Men	1.97	0.31-12.3
CVD	Black	Overall	1.14	0.70-1.87
		Women	0.90	0.46-1.79
		Men	1.46	0.72-2.96
	Non-Black	Overall	2.46	0.85-7.09
		Women	**28.7**	**2.92-282.**
		Men	0.32	0.03-2.91
CKD	Black	Overall	**1.70**	**1.01-2.86**
		Women	1.96	0.95-4.05
		Men	1.48	0.70-3.12
	Non-Black	Overall	0.74	0.18-3.05
		Women	1.45	0.27-7.76
		Men	---	

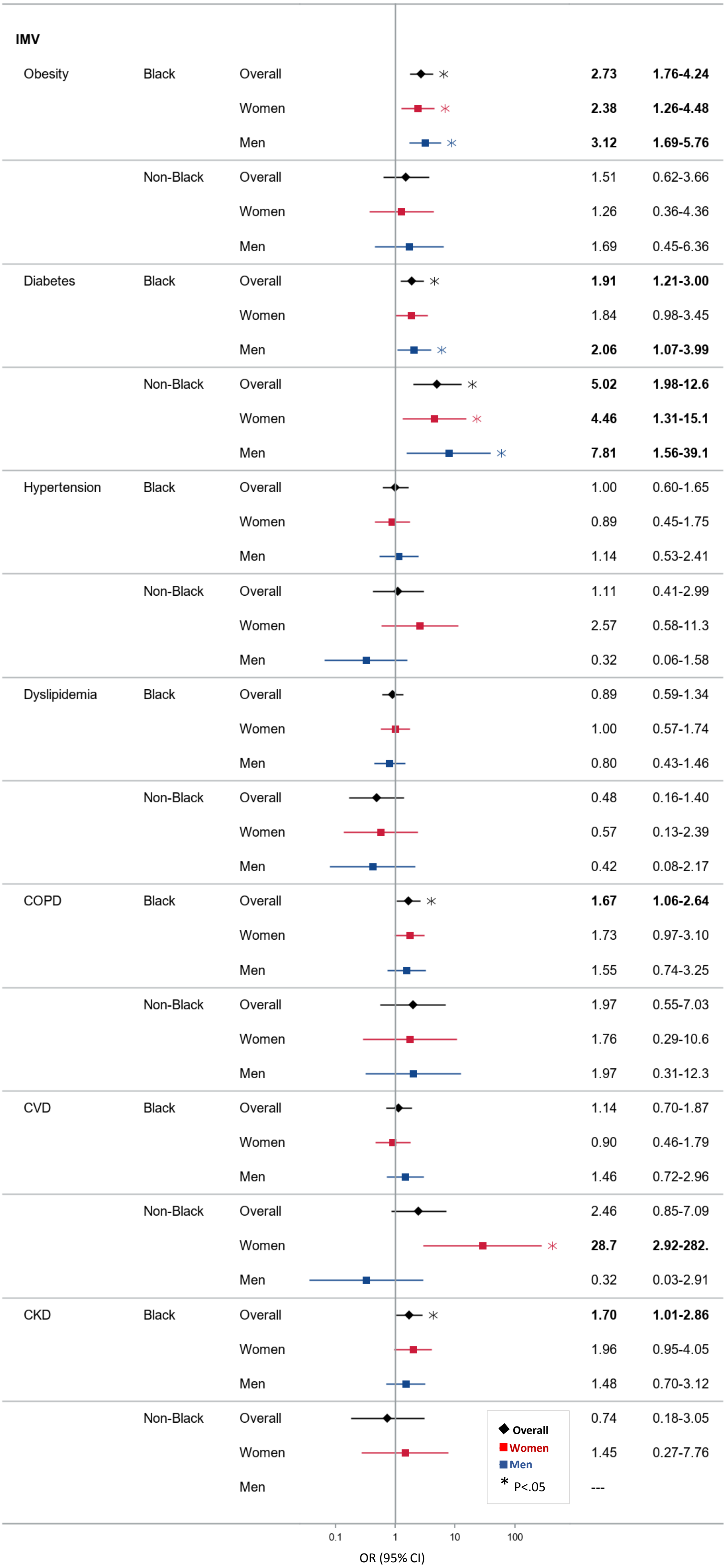

eFig 1.C Multivariable Analysis of Comorbidities and Death by Race

Parameter	Race	Sex	OR	CI
**Death**				
Obesity	Black	Overall	1.59	0.98-2.58
		Women	1.45	0.73-2.89
		Men	1.71	0.87-3.37
	Non-Black	Overall	0.75	0.28-1.96
		Women	0.51	0.13-1.93
		Men	0.96	0.2-4.60
Diabetes	Black	Overall	**1.68**	**1.00-2.81**
		Women	**2.15**	**1.01-4.55**
		Men	1.38	0.66-2.88
	Non-Black	Overall	**2.57**	**1.00-6.58**
		Women	**5.46**	**1.51-19.6**
		Men	0.73	0.13-3.94
Hypertension	Black	Overall	1.48	0.75-2.90
		Women	1.53	0.59-3.95
		Men	1.38	0.52-3.61
	Non-Black	Overall	1.36	0.44-4.17
		Women	0.99	0.20-4.78
		Men	1.35	0.24-7.54
Dyslipidemia	Black	Overall	0.94	0.59-1.50
		Women	1.08	0.56-2.06
		Men	0.84	0.42-1.66
	Non-Black	Overall	0.60	0.23-1.55
		Women	0.57	0.14-2.25
		Men	0.64	0.16-2.62
COPD	Black	Overall	1.23	0.72-2.09
		Women	1.63	0.84-3.18
		Men	0.75	0.30-1.88
	Non-Black	Overall	0.72	0.17-2.96
		Women	0.44	0.04-4.63
		Men	0.91	0.14-5.90
CVD	Black	Overall	1.51	0.89-2.56
		Women	1.01	0.48-2.14
		Men	**2.29**	**1.08-4.87**
	Non-Black	Overall	1.70	0.64-4.51
		Women	3.35	0.81-13.7
		Men	0.76	0.15-3.73
CKD	Black	Overall	**2.26**	**1.30-3.93**
		Women	**2.46**	**1.13-5.35**
		Men	2.06	0.94-4.51
	Non-Black	Overall	1.89	0.64-5.58
		Women	2.61	0.60-11.3
		Men	0.70	0.11-4.25

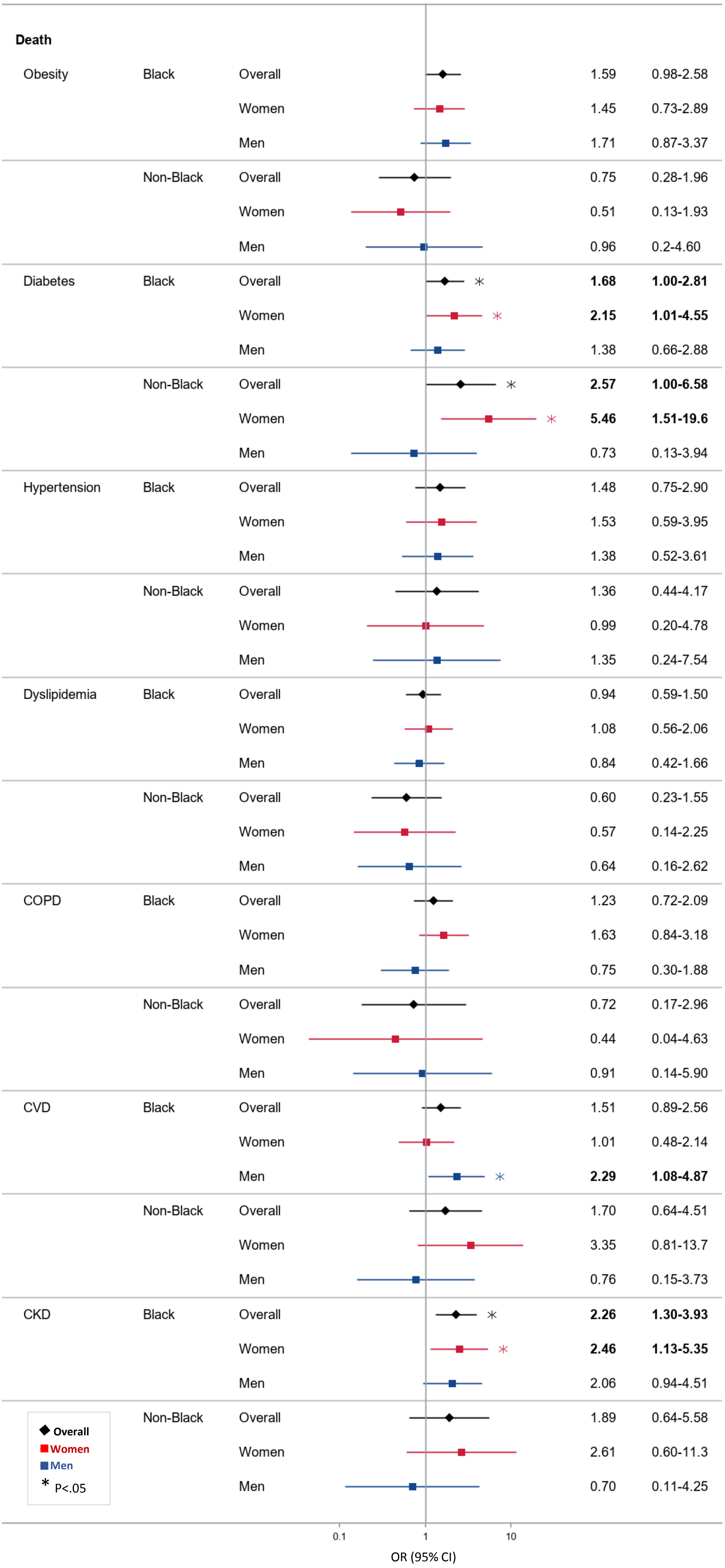

eFig 2.A Multivariable Analysis of Biomarkers and ICU by Race

Parameter	Race	Sex	OR	CI
**ICU**				
CRP (>41.2 mg/dL)	Black	Overall	**3.29**	**1.94-5.56**
		Women	**3.05**	**1.51-6.13**
		Men	**3.77**	**1.68-8.45**
	Non-Black	Overall	**4.47**	**1.57-12.7**
		Women	**5.91**	**1.43-24.4**
		Men	3.46	0.71-16.9
D-Dimer (>1.5 mg/L)	Black	Overall	1.49	0.80-2.76
		Women	0.63	0.27-1.42
		Men	**6.17**	**2.04-18.6**
	Non-Black	Overall	**4.45**	**1.37-14.4**
		Women	2.84	0.69-11.6
		Men	**12.7**	**1.2-134.**
Ferritin (>300 ng/mL)	Black	Overall	**2.50**	**1.57-3.96**
		Women	**2.42**	**1.37-4.27**
		Men	**3.20**	**1.36-7.51**
	Non-Black	Overall	1.78	0.78-4.04
		Women	2.28	0.78-6.67
		Men	1.34	0.38-4.77
LDH (>365 U/L)	Black	Overall	**3.73**	**2.47-5.63**
		Women	**4.02**	**2.31-7.00**
		Men	**3.70**	**1.95-7.02**
	Non-Black	Overall	**4.83**	**2.02-11.5**
		Women	**8.43**	**2.33-30.5**
		Men	3.11	0.9-10.8
Procalcitonin (>0.5 ng/mL)	Black	Overall	**2.44**	**1.46-4.08**
		Women	2.04	0.94-4.41
		Men	**2.86**	**1.41-5.80**
	Non-Black	Overall	**3.45**	**1.16-10.2**
		Women	**8.23**	**1.37-49.3**
		Men	1.79	0.36-8.84
Troponin (>0.1 ng/mL)	Black	Overall	**1.80**	**1.00-3.22**
		Women	1.51	0.66-3.45
		Men	2.25	0.97-5.21
	Non-Black	Overall	2.07	0.68-6.31
		Women	1.31	0.29-5.89
		Men	5.54	0.81-37.9
NT-Pro BNP (>400 pg/mL)	Black	Overall	**2.66**	**1.51-4.66**
		Women	1.89	0.89-4.01
		Men	**4.17**	**1.75-9.96**
	Non-Black	Overall	1.76	0.65-4.78
		Women	4.74	0.96-23.2
		Men	0.66	0.14-3.06
Lymphocytes (<1*10^3/uL)	Black	Overall	1.16	0.79-1.68
		Women	1.53	0.92-2.54
		Men	0.85	0.48-1.48
	Non-Black	Overall	1.53	0.73-3.22
		Women	1.64	0.54-4.95
		Men	1.60	0.55-4.61
NLR (>6)	Black	Overall	**2.01**	**1.38-2.93**
		Women	**2.28**	**1.33-3.91**
		Men	**1.83**	**1.07-3.15**
	Non-Black	Overall	**3.31**	**1.57-6.99**
		Women	**4.45**	**1.60-12.3**
		Men	2.64	0.87-8.00

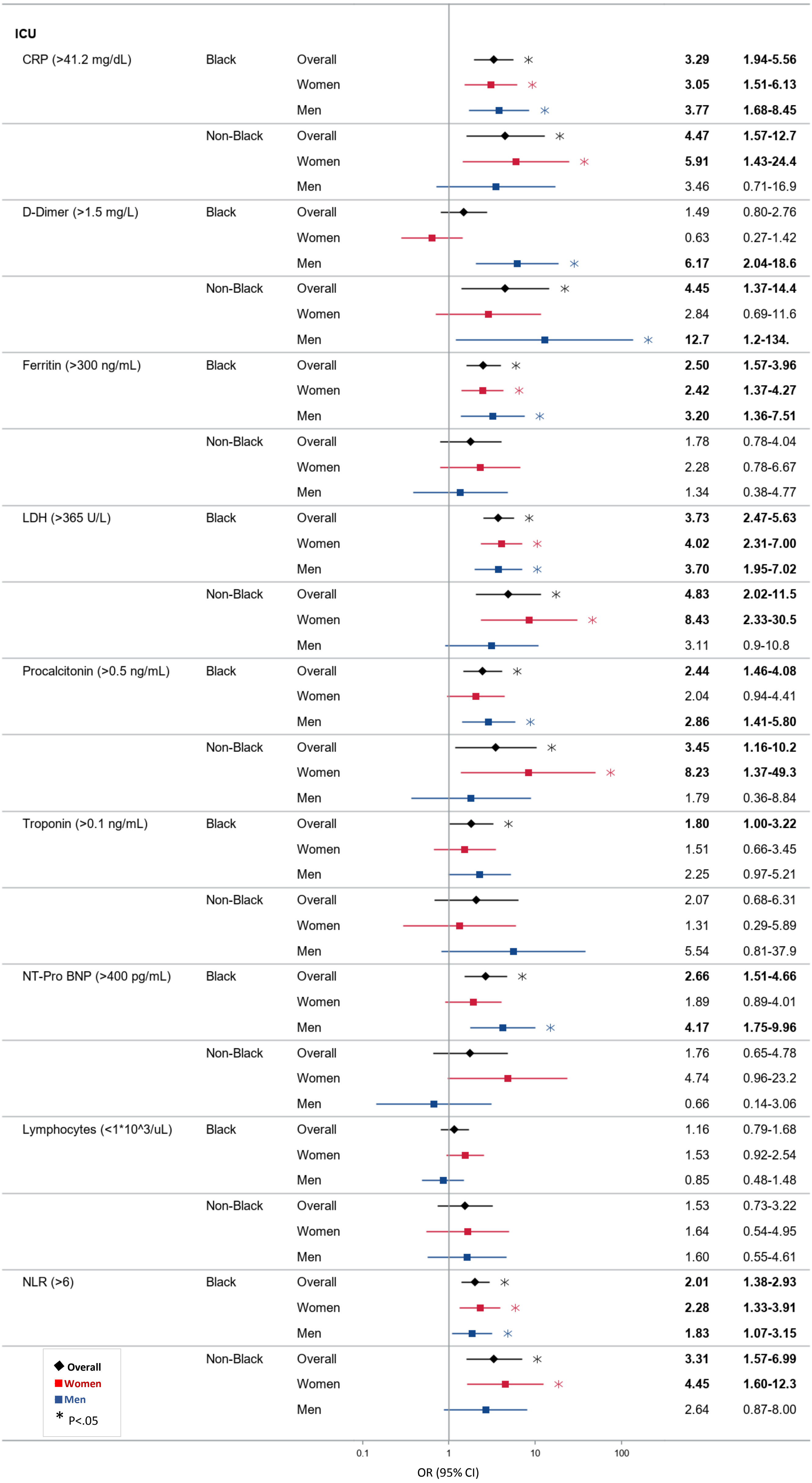

◆ Overall
■ Women
■ Men
* P<.05

eFig 2.B Multivariable Analysis of Biomarkers and IMV by Race

Parameter	Race	Sex	OR	CI
**IMV**				
CRP (>41.2 mg/dL)	Black	Overall	**3.45**	**1.87-6.35**
		Women	**4.70**	**1.91-11.5**
		Men	**2.47**	**1.05-5.76**
	Non-Black	Overall	**6.45**	**1.40-29.7**
		Women	**8.83**	**1.05-74.0**
		Men	3.40	0.38-30.3
D-Dimer (>1.5 mg/L)	Black	Overall	1.21	0.63-2.34
		Women	0.58	0.25-1.35
		Men	**4.52**	**1.36-14.9**
	Non-Black	Overall	2.46	0.62-9.74
		Women	0.97	0.21-4.37
		Men	----	
Ferritin (>300 ng/mL)	Black	Overall	**2.55**	**1.51-4.30**
		Women	**2.39**	**1.28-4.47**
		Men	**3.44**	**1.25-9.42**
	Non-Black	Overall	1.71	0.62-4.69
		Women	2.68	0.73-9.78
		Men	0.68	0.14-3.29
LDH (>365 U/L)	Black	Overall	**4.22**	**2.71-6.58**
		Women	**4.42**	**2.44-8.02**
		Men	**4.55**	**2.27-9.12**
	Non-Black	Overall	**3.43**	**1.19-9.89**
		Women	**4.38**	**1.16-16.4**
		Men	3.18	0.47-21.4
Procalcitonin (>0.5 ng/mL)	Black	Overall	**2.56**	**1.49-4.39**
		Women	**2.83**	**1.25-6.41**
		Men	**2.40**	**1.16-4.95**
	Non-Black	Overall	**4.98**	**1.40-17.6**
		Women	4.47	0.74-26.9
		Men	5.68	0.78-41.3
Troponin (>0.1 ng/mL)	Black	Overall	1.39	0.76-2.56
		Women	1.19	0.5-2.84
		Men	1.72	0.72-4.07
	Non-Black	Overall	3.24	0.92-11.4
		Women	2.23	0.41-11.9
		Men	5.10	0.61-42.6
NT-Pro BNP (>400 pg/mL)	Black	Overall	**2.21**	**1.25-3.89**
		Women	1.64	0.76-3.50
		Men	**3.31**	**1.39-7.84**
	Non-Black	Overall	1.79	0.58-5.53
		Women	4.82	0.91-25.4
		Men	0.61	0.09-3.91
Lymphocytes (<1*10^3/uL)	Black	Overall	0.93	0.62-1.40
		Women	1.10	0.63-1.91
		Men	0.76	0.41-1.4
	Non-Black	Overall	1.58	0.65-3.87
		Women	1.90	0.55-6.51
		Men	1.06	0.27-4.17
NLR (>6)	Black	Overall	**1.52**	**1.01-2.28**
		Women	**1.80**	**1.01-3.21**
		Men	1.36	0.76-2.43
	Non-Black	Overall	**3.75**	**1.48-9.50**
		Women	**5.28**	**1.58-17.6**
		Men	2.25	0.52-9.67

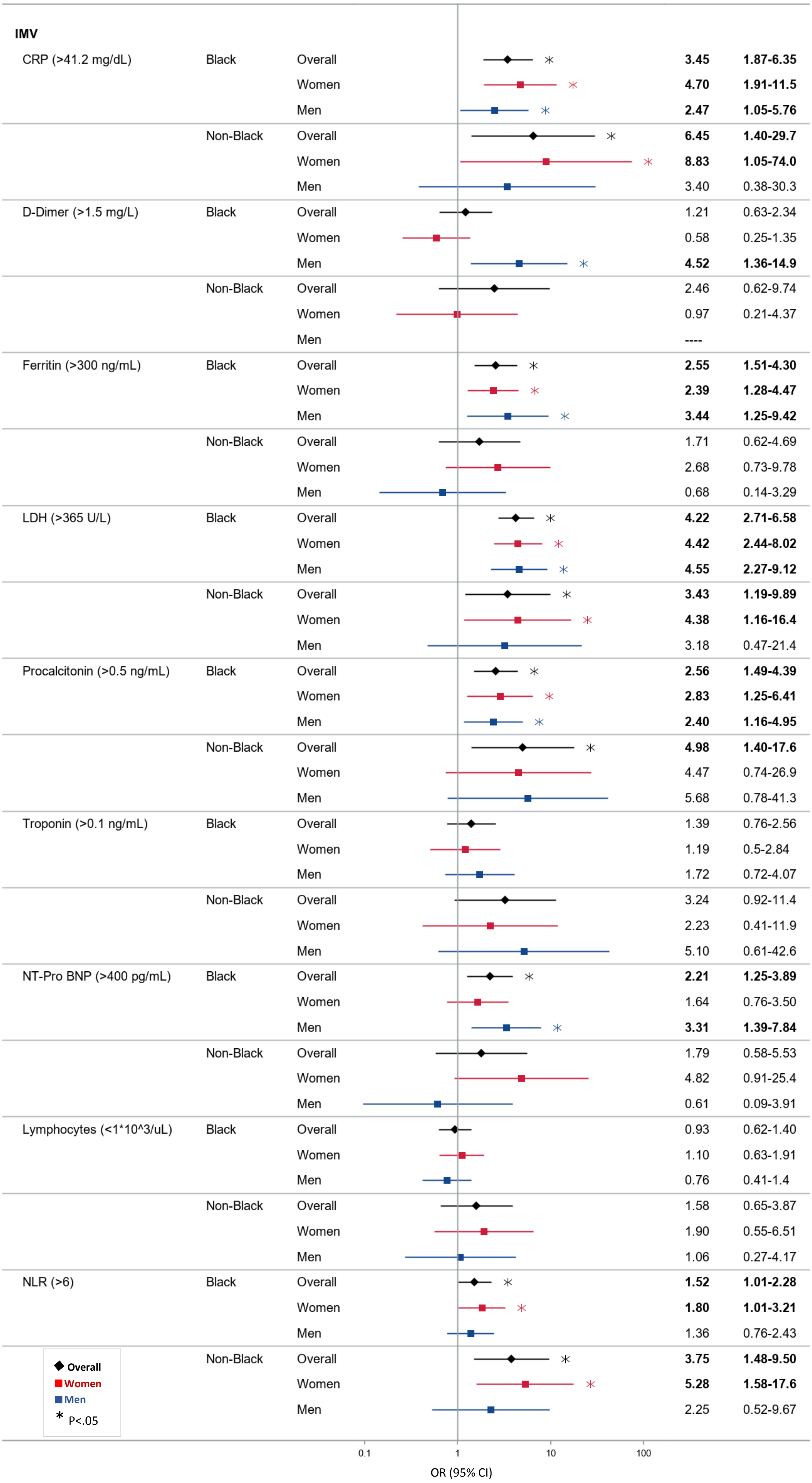

◆ Overall
■ Women
■ Men
* P<.05

eFig 2.C Multivariable Analysis of Biomarkers and Death by Race

Parameter	Race	Sex	OR	CI
**Death**				
CRP (>41.2 mg/dL)	Black	Overall	**5.15**	**2.15-12.3**
		Women	**5.53**	**1.63-18.7**
		Men	**4.90**	**1.39-17.2**
	Non-Black	Overall	**6.04**	**1.58-23.0**
		Women	2.53	0.44-14.5
		Men	**13.1**	**1.45-118.**
D-Dimer (>1.5 mg/L)	Black	Overall	1.16	0.51-2.62
		Women	0.73	0.26-1.98
		Men	3.02	0.67-13.5
	Non-Black	Overall	4.08	0.90-18.4
		Women	1.80	0.32-10.0
		Men	**34.0**	**1.53-757.**
Ferritin (>300 ng/mL)	Black	Overall	**3.01**	**1.55-5.83**
		Women	**4.37**	**1.75-10.8**
		Men	1.83	0.69-4.86
	Non-Black	Overall	1.26	0.48-3.30
		Women	2.20	0.59-8.20
		Men	0.42	0.09-1.94
LDH (>365 U/L)	Black	Overall	**4.79**	**2.78-8.26**
		Women	**8.67**	**3.94-19.0**
		Men	**2.66**	**1.21-5.87**
	Non-Black	Overall	1.58	0.52-4.76
		Women	1.96	0.44-8.64
		Men	1.15	0.21-6.19
Procalcitonin (>0.5 ng/mL)	Black	Overall	**4.28**	**2.31-7.91**
		Women	**5.49**	**2.22-13.5**
		Men	**3.61**	**1.55-8.37**
	Non-Black	Overall	**4.06**	**1.17-14.0**
		Women	2.49	0.43-14.4
		Men	5.52	0.79-38.1
Troponin (>0.1 ng/mL)	Black	Overall	**2.60**	**1.40-4.84**
		Women	2.16	0.9-5.18
		Men	**3.22**	**1.32-7.83**
	Non-Black	Overall	**5.78**	**1.65-20.1**
		Women	2.52	0.47-13.4
		Men	**23.1**	**1.71-312.**
NT-Pro BNP (>400 pg/mL)	Black	Overall	**3.64**	**1.95-6.81**
		Women	**2.68**	**1.15-6.23**
		Men	**5.56**	**2.12-14.5**
	Non-Black	Overall	2.46	0.76-7.99
		Women	**6.34**	**1.07-37.3**
		Men	0.89	0.14-5.41
Lymphocytes (<1*10^3/uL)	Black	Overall	0.91	0.57-1.46
		Women	0.74	0.38-1.43
		Men	1.13	0.56-2.26
	Non-Black	Overall	1.60	0.65-3.90
		Women	1.57	0.44-5.60
		Men	1.20	0.31-4.55
NLR (>6)	Black	Overall	**1.77**	**1.12-2.82**
		Women	**2.43**	**1.25-4.70**
		Men	1.37	0.70-2.66
	Non-Black	Overall	**2.74**	**1.11-6.74**
		Women	3.00	0.89-10.1
		Men	2.46	0.62-9.75

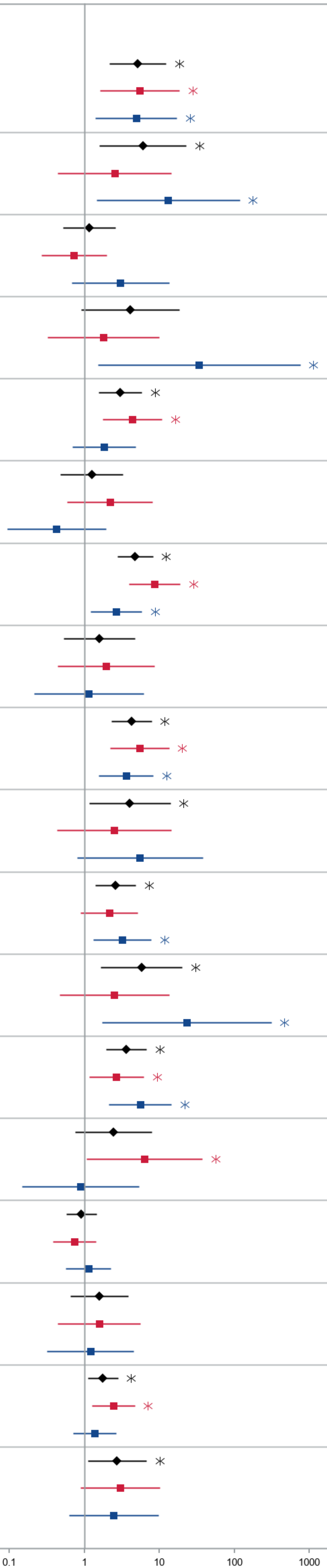

◆ Overall
■ Women
■ Men
* P<.05

eTable 1. Demographic Characteristics and comorbidities prior to admission– Blacks vs. non-Blacks

	**Black**				**Non-black**			
	**All**	**Women**	**Men**	**P value**	**All**	**Women**	**Men**	**P value**
**Age, mean (SD), years**	59.7 (14.9)	60.2 (15.2)	59.5 (14.3)	0.6	62.9 (19.1)	65.7 (20.7)	60.4 (17.4)	0.06
	**N (%)**	**N (%)**	**N (%)**		**N (%)**	**N (%)**	**N (%)**	
**Sex**	**---**	315 (54.7)	261 (45.3)	**0.02**	---	91 (46.9)	103 (53.1)	0.74
**Hospital site**				0.6				0.62
Tulane	221 (38)	124 (39.4)	97 (37.2)		76 (39.2)	34 (37.4)	42 (40.9)	
UMC [a]	360 (62)	191 (60.6)	164 (62.8)		118 (60.8)	57 (62.6)	61 (59.1)	
**Comorbidity**								
Obesity	326 (56.3)	211 (67.2)	114 (43.9)	**<0.0001**	82 (42.9)	46 (51.7)	36 (35.3)	0.02
BMI category (kg/m^2)[b]				**<0.0001**				0.09
BMI<25	106 (18.3)	44 (14)	60 (23.1)		58 (30.4)	25 (28)	33 (32.3)	
25≤BMI<30	147 (25.4)	59 (18.8)	86 (33.1)		51 (26.7)	18 (20.2)	33 (32.3)	
30≤BMI<35	122 (21.1)	73 (23.3)	49 (18.8)		34 (17.8)	16 (18)	18 (17.6)	
35≤BMI<40	93 (16)	61 (19.4)	32 (12.3)		25 (13.1)	15 (16.9)	10 (9.8)	
BMI≥40	111 (19.2)	77 (24.5)	33 (12.7)		23 (12)	15 (16.9)	8 (7.8)	
Diabetes	223 (38.4)	128 (40.6)	93 (35.6)	0.22	50 (25.8)	27 (29.7)	23 (22.3)	0.24
Hypertension	455 (78.3)	250 (79.4)	203 (77.8)	0.6	117 (60.3)	65 (71.4)	52 (50.5)	0.003
Dyslipidemia	239 (41.1)	134 (42.5)	104 (39.9)	0.5	52 (16.8)	24 (26.4)	28 (27.2)	0.9
Chronic obstructive pulmonary disease	81 (13.9)	48 (15.2)	31 (11.9)	0.24	15 (7.7)	8 (8.8)	7 (6.8)	0.6
Asthma	77 (13.3)	55 (17.5)	22 (8.4)	**0.001**	6 (3.1)	3 (3.3)	3 (2.9)	0.9
Cerebrovascular disease	76 (13.5)	43 (14)	33 (13.2)	0.79	18 (10)	8 (9.3)	10 (10.6)	0.77
Cardiovascular disease	123 (21.9)	68 (22.1)	55 (22)	0.98	31 (17.2)	16 (18.6)	15 (16)	0.64
Heart failure	87 (15.5)	49 (15.9)	38 (15.2)	0.81	22 (12.2)	13 (15.1)	9 (9.6)	0.25
Myocardial infarction	37 (6.6)	17 (5.5)	20 (8)	0.24	9 (5)	3 (3.5)	6 (6.4)	0.37
Peripheral vascular disease	21 (3.7)	15 (4.9)	6 (2.4)	0.12	4 (2.2)	1 (1.2)	3 (3.2)	0.35
Chronic kidney disease	104 (18.5)	48 (15.6)	56 (22.4)	**0.03**	22 (12.2)	14 (16.3)	8 (8.5)	0.11
Chronic liver disease	28 (4.8)	8 (3.33)	19 (9.22)	**0.009**	8 (4.1)	3 (3.3)	5 (4.9)	0.58
Dementia	25 (4.4)	15 (4.9)	9 (3.6)	0.46	30 (16.7)	19 (22.1)	11 (11.7)	0.06
**Charlson index, mean (SD)**	3.78 (2.7)	3.7 (2.6)	3.9 (2.7)	0.34	3.6 (3)	4.1 (3)	3.1 (2.8)	**0.02**

[a] UMC: University Medical Center New Orleans; [b] BMI: Body Mass Index.

eTable 2. Clinical symptoms and biomarkers at admission – Blacks vs. non-Blacks

	**Black**			**P**	**Non-black**			**P**
	**All (N [%])**	**Women (N [%])**	**Men (N [%])**		**All (N [%])**	**Women (N [%])**	**Men (N [%])**	
**Clinical symptoms**								
Fever	338 (60.5)	182 (60.1)	153 (61)	0.83	98 (56)	41 (47.7)	57 (64)	0.03
Digestive symptoms (nausea, vomiting or diarrhea)	220 (39.4)	132 (43.6)	84 (33.5)	0.01	49 (28)	21 (24.4)	28 (31.5)	0.3
Cough (non-productive)	250 (44.7)	150 (49.5)	98 (39)	0.01	79 (45.1)	34 (39.5)	45 (50.6)	0.14
Dyspnea	326 (58.3)	193 (63.7)	132 (52.6)	0.008	95 (54.3)	45 (52.3)	50 (56.)2	0.61
Myalgia	155 (27.8)	91 (30.1)	63 (25.1)	0.19	47 (26.9)	24 (27.9)	23 (25.8)	0.75
Fatigue	179 (32.1)	108 (35.6)	70 (28)	0.05	57 (32.6)	31 (36.1)	26 (29.2)	0.33
**Biomarkers**								
ALT >56 U/L [a]	149 (26.9)	55 (18.1)	92 (37.4)	**<0.0001**	55 (31.8)	25 (29.4)	30 (34.1)	0.51
AST >40 U/L [b]	150 (27.1)	62 (20.5)	86 (35)	**0.0001**	42 (24.4)	14 (16.5)	28 (32.2)	**0.01**
CRP >3mg/L [c]	483 (98.8)	267 (98.9)	213 (98.6)	0.78	148 (98)	71 (98.6)	77 (97.5)	0.61
D-Dimer >0.5 mg/L	368 (90.4)	198 (88.3)	167 (92.8)	0.14	117 (88)	56 (90.3)	61 (85.9)	0.43
Ferritin >300 ng/mL	341 (70.3)	171 (62.6)	167 (79.9)	**<0.0001**	96 (62.3)	38 (52.1)	58 (71.6)	**0.01**
Glucose ≥140 mg/dL	235 (50.7)	128 (49.4)	104 (51.5)	0.66	51 (41.1)	25 (40.3)	26 (41.9)	0.86
A1c >5.7%	209 (83.3)	107 (82.3)	101 (84.2)	0.69	52 (85.6)	24 (88.9)	28 (8.4)	0.47
LDH ≥220 U/L [d]	415 (86.3)	184 (83.6)	155 (87.6)	0.27	107 (72.8)	52 (74.3)	55 (71.4)	0.7
Procalcitonin ≥0.1 ng/mL	334 (86.1)	177 (82.7)	156 (90.2)	**0.03**	89 (84)	42 (84)	47 (83.9)	0.99
Troponin ≥0.04 ng/ml	143 (30.4)	66 (25.7)	74 (35.2)	0.02	34 (25.8)	19 (27.1)	15 (24.2)	0.7
NT-Pro BNP >400 pg/ml [e]	85 (20.1)	49 (21)	36 (19.1)	0.61	36 (28.8)	20 (35.1)	16 (23.5)	0.15
WBC count <4.0*10^3/uL [f]	55 (10)	27 (8.9)	28 (11.3)	0.33	16 (9.1)	8 (9.3)	8 (9)	0.94
Lymphocytes <1.0*10^3/uL	236 (42.4)	116 (38)	118 (47.8)	**0.02**	68 (39.1)	29 (34.1)	39 (43.8)	0.19
Platelets <150*10^3/uL	83 (14.9)	40 (13.1)	43 (17.4)	0.16	37 (21.1)	16 (18.6)	21 (23.6)	0.42
Neutrophils >1.8*10^3/uL	540 (97)	294 (96.4)	241 (97.6)	0.42	168 (96.6)	82 (96.5)	86 (96.6)	0.95
NLR>6 [g]	197 (35.4)	93 (30.5)	101 (40.9)	**0.01**	78 (44.8)	32 (37.7)	46 (51.7)	0.06
Monocytes >1.0*10^3/uL	67 (12)	27 (8.9)	38 (15.4)	**0.01**	36 (20.7)	15 (17.7)	21 (23.6)	**0.33**

[a] ALT: Alanine Aminotransferase; [b] AST: Aspartate Aminotransferase; [c] CRP: C-reactive protein; [d] LDH: lactate dehydrogenase; [e] NT-Pro BNP: N-terminal pro b-type natriuretic peptide; [f] WBC: White blood cell counts [g] NLR: neutrophil-to-lymphocyte ratio
